# Supplementary material for: The genome sequence of the Bird-cherry Ermine moth, Yponomeuta evonymella (Linnaeus, 1758)
Source: Wellcome Open Res. 2024 Dec 16;9:618. Originally published 2024 Oct 21. [Version 2] doi: 10.12688/wellcomeopenres.23211.2 (PMC11809182; doi:10.12688/wellcomeopenres.23211.2)
Supplement: Peer review history

# Reviewer report: Reviewer response for version 2

**DOI:** 10.21956/wellcomeopenres.25959.r115741

**Published:** 2024-12-19

**Authors:** Yu-Feng Huang

**Affiliations:** 1 National Chung Hsing University, Taichung, Taiwan

The authors have revised the manuscript following the suggestions and the manuscript is now ready for indexing. I have no further comments.

Are sufficient details of methods and materials provided to allow replication by others?

Yes

Is the rationale for creating the dataset(s) clearly described?

Partly

Are the datasets clearly presented in a useable and accessible format?

Yes

Are the protocols appropriate and is the work technically sound?

Partly

Reviewer Expertise:

bioinformatics, genome assembly, annotation and evaluation, mitochondrial genome, cancer bioinformatics,

I confirm that I have read this submission and believe that I have an appropriate level of expertise to confirm that it is of an acceptable scientific standard.

# Reviewer report: Reviewer response for version 2

**DOI:** 10.21956/wellcomeopenres.25959.r115740

**Published:** 2024-12-17

**Authors:** Stephan Koblmüller

**Affiliations:** 1 University of Graz, Graz, Austria

After modifying the text in the background section following my suggestions, the manuscript is now ready for publication (at least in my view). I have no further comments.

Are sufficient details of methods and materials provided to allow replication by others?

Yes

Is the rationale for creating the dataset(s) clearly described?

Yes

Are the datasets clearly presented in a useable and accessible format?

Yes

Are the protocols appropriate and is the work technically sound?

Yes

Reviewer Expertise:

phylogenetics-/genomics, population genetics, molecular biodiversity research

I confirm that I have read this submission and believe that I have an appropriate level of expertise to confirm that it is of an acceptable scientific standard.

# Reviewer report: Reviewer response for version 2

**DOI:** 10.21956/wellcomeopenres.25959.r113721

**Published:** 2024-12-17

**Authors:** Jay K Goldberg

**Affiliations:** 1 Department of Crop Genetics, John Innes Centre, Norwich, England, UK

The article describes the production of a de novo genome assembly for the bird-cherry Ermine moth. As is usually the case for ToL genomes, the dataset itself is of high quality and will be useful for researchers interested in insect genomics. I have only a few minor comments:

**Background:**

 - Generic names should not be abbreviated when they come at the beginning of a sentence.

 - Given the species' large geographic range, it would be worth mentioning any past research on population structure and/or the presence of any subspecies.

Are sufficient details of methods and materials provided to allow replication by others?

Yes

Is the rationale for creating the dataset(s) clearly described?

Yes

Are the datasets clearly presented in a useable and accessible format?

Yes

Are the protocols appropriate and is the work technically sound?

Yes

Reviewer Expertise:

entomology, genomics, evolution, plant-insect interactions

I confirm that I have read this submission and believe that I have an appropriate level of expertise to confirm that it is of an acceptable scientific standard.

# Reviewer report: Reviewer response for version 1

**DOI:** 10.21956/wellcomeopenres.25563.r113727

**Published:** 2024-12-11

**Authors:** Stephan Koblmüller

**Affiliations:** 1 University of Graz, Graz, Austria

In this data note, Boyes *et al*. present the genome assembly of the Bird-cherry Ermine moth, a yponeumatid species widely distributed n the Palaearctic. The background section gives a concise, but nonetheless informative, introduction to the study species. I have only some minor comments about the background section:

 1. The last two paragraphs in the background section are somewhat redundant. Please omit one of these two paragraphs.

 2. I suggest to slightly rephrase these paragraphs anyway as the reference genome of this species per se will refine neither our understanding of phylogenetic relationships nor pheromone production and insect-host relationships. Here, additional data would be required. 

 The data are clear, and the methods are well described. I have no objections to any of the data presented or the results. As far as I can tell, the datasets are deposited in publicly accessible repositories.

Are sufficient details of methods and materials provided to allow replication by others?

Yes

Is the rationale for creating the dataset(s) clearly described?

Yes

Are the datasets clearly presented in a useable and accessible format?

Yes

Are the protocols appropriate and is the work technically sound?

Yes

Reviewer Expertise:

phylogenetics-/genomics, population genetics, molecular biodiversity research

I confirm that I have read this submission and believe that I have an appropriate level of expertise to confirm that it is of an acceptable scientific standard, however I have significant reservations, as outlined above.

# Reviewer report: Reviewer response for version 1

**DOI:** 10.21956/wellcomeopenres.25563.r108131

**Published:** 2024-11-18

**Authors:** Yu-Feng Huang

**Affiliations:** 1 National Chung Hsing University, Taichung, Taiwan

This manuscript describes the sequencing and assembly of the genome of *Yponomeuta evonymella* using advanced sequencing technologies, including HiFi long reads and Hi-C scaffolding. The study provides a high-quality genome assembly, offering valuable insights into the genomic architecture of a Lepidopteran species with significant ecological and evolutionary importance. This work represents an important contribution to the field of Lepidoptera genomics, with broad implications for phylogenetic studies and functional genomics. However, there are some areas that require further attention:

 1. Genome Evaluation:

 • The manuscript should go beyond listing the computational tools used in the pipeline by providing detailed quality metrics and the rationale for the final evaluation. These metrics should include genome completeness scores, k-mer analysis, error rates, and contamination screening results. Additionally, comparing these metrics with related species would add valuable context and further validate the quality of the assembly.

 2. Scope Beyond Genome Assembly:

 • While the primary focus of the journal is on presenting genome assemblies, the manuscript would benefit from discussing functional and ecological insights derived from the assembly. In particular:

 • Highlight key genes associated with mimicry, pheromone production, or host-plant interactions, as these are likely to have ecological or evolutionary relevance.

 • Present any unique genomic features or lineage-specific adaptations identified in the assembly.

 By addressing these points, the manuscript will provide a more comprehensive evaluation of the genome and enhance its relevance to broader ecological and evolutionary research.

Are sufficient details of methods and materials provided to allow replication by others?

Yes

Is the rationale for creating the dataset(s) clearly described?

Partly

Are the datasets clearly presented in a useable and accessible format?

Yes

Are the protocols appropriate and is the work technically sound?

Partly

Reviewer Expertise:

bioinformatics, genome assembly, annotation and evaluation, mitochondrial genome, cancer bioinformatics,

I confirm that I have read this submission and believe that I have an appropriate level of expertise to confirm that it is of an acceptable scientific standard, however I have significant reservations, as outlined above.
